# The Pathology and Treatment of Yellow Fellow Fever; with Some Remarks upon the Nature of Its Cause and Its Prevention

**Published:** 1881-05

**Authors:** H. D. Schmidt

**Affiliations:** Pathologist of the Charity Hospital of New Orleans; Member of the Academy of the Natural Sciences of Philadelphia; of the American Neurological Association; of the American Association for the Advancement of Science; Honorary Member of the New Orleans Medical and Surgical Association, Etc., Etc.


### PATHOLOGY AND TREATMENT

OF

### YELLOW FEVER;

WITH SOME REMARKS UPON

### THE NATURE OF ITS CAUSE AND ITS PREVENTION.

BY

H. D. SCHMIDT, M.D.,

PATHOLOGIST OF THE CHARITY HOSPITAL OF NEW ORLEANS; MEMBER OF THE ACADEMY OF THE NATURAL SCIENCES OF PHILADELPHIA; OF THE AMERICAN NEUROLOGICAL ASSOCIATION; OF THE AMERICAN ASSOCIATION FOR THE ADVANCEMENT OF SCIENCE; HONORARY MEMBER OF THE NEW ORLEANS MEDICAL AND SURGICAL ASSOCIATION, ETC., ETC.

With Five Illustrative Plates.

CHICAGO:
THE CHICAGO MEDICAL PRESS ASSOCIATION.
1881.

Entered according to Act of Congress, in the year 1881, by H. D. SCHMIDT, M.D., in the office of the Librarian of Congress, at Washington, D. C.

This Work I Dedicate

TO MY DISTINGUISHED PRECEPTOR AND FRIEND,

PROF. JOSEPH LEIDY, M.D.,

OF THE UNIVERSITY OF PENNSYLVANIA,

WHO FIRST TAUGHT ME THE PRINCIPLES OF EXACT SCIENCE,

AND WHOSE SYMPATHY AND KINDNESS

HAVE BEEN TO ME,

IN ALL THE CONJUNCTURES OF LIFE,

A NEVER-FAILING

Solace and Support.

### PREFACE.

In preparing this Treatise on Yellow Fever, the object I have kept steadily in view, has been to draw as distinct and correct a sketch of its various pathological phenomena, and their mutual relations, as our present knowledge of the disease permits.

Though I indulge the hope that my purpose has been to some extent accomplished, I am not blind to the numerous and important gaps which are left, to be filled, hereafter, by further systematic investigations. But imperfect as the present work must, of necessity, be, I trust it will serve as a guide to the practicing physician in his efforts to master this most malignant disease.

While the observations of the clinical phenomena were chiefly made at the bedside of my private patients during several epidemics, the pathological studies were pursued, for the most part, in the dead-house and pathological laboratory of the Charity Hospital of this city; an institution which, for the study of yellow fever, probably offers more favorable opportunities than any other situated in the yellow fever zone.

Having on several occasions been disappointed by lithographers making inaccurate copies of my original pencil-drawings, I have made the attempt to lithograph the illustrations accompanying this work, upon the stone myself. Though they may not be finished as exquisitely as by the hand of a first-class professional artist, I have, at least, the satisfaction of knowing that they represent the tissues, accurately, as I observed them under the microscope.

NEW ORLEANS, LA., May, 1880.

### CONTENTS.

INTRODUCTION..........................................................

CLINICAL PHENOMENA, OBSERVED DURING THE COURSE OF THE DISEASE..........................................................

PATHOLOGICAL ANATOMY:

1. Condition of the Surface and Internal Organs of the Body, as revealed by the Autopsy..........................................

2. Pathological Changes, occurring in the Tissues and Organs during the Course of the Disease, and revealed by Microscopical Examination..........................................

GENERAL PATHOLOGY..........................................................

REMARKS ON THE PROBABLE NATURE OF THE INFECTIOUS POISON OF YELLOW FEVER..........................................................

TREATMENT..........................................................

REMARKS ON THE PREVENTION OF YELLOW FEVER..........................................

### INTRODUCTION.

There are few infectious diseases which have engaged the interest of medical men as much as yellow fever. In consequence, the literature of this disease, beginning in the seventeenth century and extending to our time, has assumed very considerable dimensions.* But extensive as this literature appears, it has, in reality, but little advanced our knowledge of the true pathology of this disease, for the reason that the authors, especially the older ones, had contented themselves with basing their pathological theories chiefly upon the clinical phenomena observed at the bedside, or, at the utmost, upon the macroscopical examinations accompanying the autopsies, which, as may be presumed, were not always made in the systematic style and manner essential to an

* The works and monographs treating upon yellow fever, and composing the Bibliography of Dr. *La Roche's* great work on this disease, *alone*, amount to *nine hundred and seventy-five.*

accurate scientific investigation. Accordingly, the authors principally confined their writings to a description of the history of the disease, or to the observations which they made at the bedside, and from which they formed their various speculative theories as to its probable cause.

From these remarks, however, it must not be inferred that our predecessors were inferior students or observers, for their clinical observations have in most points been corroborated by our own; their deficiency of observation was only due to want of acquaintance with those important pathological facts, elicited chiefly by the aid of the microscope in more recent times; for, twenty-five years have hardly elapsed since this instrument was first used to demonstrate the structural changes taking place in various organs during the course of yellow fever. In judging, therefore, of the merits of our predecessors, the rapid progress made in our own time in human physiology and pathology, upsetting many of the older theories, must be taken into consideration. Thus, the former theories of the processes of fever and inflammation have been placed upon different bases, greatly by the labors of Virchow. The febrile process was still shrouded in mystery, until, not many years ago, by numerous experiments relating to the interchanges of matter and the production of heat in the blood during this process, more light was thrown upon it. With the knowledge of modern pathology, therefore, the nature of yellow fever should now be more successfully studied, and means be devised to mitigate, at least, the disease, and deprive it of its terror. The systematic and thorough labors of European pathologists into the pathology and treatment of small-pox, typhus and typhoid fever, diphtheria, and other infectious diseases, may serve as an example. The late epidemics at New Orleans and Memphis have furnished sufficient material for such studies; and though we earnestly hope that these calamities will not be witnessed again, we must presume that sporadic cases of yellow fever will always occur to serve for more thorough and systematic studies than have hitherto been made.

The future studies into the pathology of yellow fever, however, in order to be productive, should be made with sufficient accuracy and system, as otherwise they could only give rise to false impressions and confusion. The clinical phenomena of the individual cases, particularly the comparative state of the pulse and temperature, the gastric and nervous phenomena, the apparent condition of the bowels and bladder, and the frequency and character of the discharges from the latter organs should be closely observed and carefully noted, in order to be accurately compared with the pathological changes met with in the various organs after death. It is only from investigation of this kind, systematically pursued with the necessary accuracy of observation by trustworthy and experienced pathologists, who are, besides, thoroughly acquainted with the normal structure of the organs, that additional facts may be elicited. Such labors, however, are not as easily and as readily performed as is imagined. The possession and the knowledge of handling a microscope do not suffice for the correct recognition and determination of pathological changes, nor do they qualify every microscopic operator for labors as important as these; for, besides the experience essential to an accurate examination, the microscopist should also possess the necessary knowledge and experience in the proper preparation of the tissues, in order to preserve their integrity, or render them otherwise fit for microscopical examination. This knowledge is only possessed by a comparatively small number of those who use the microscope. Besides this, the investigator should possess a free and unbiased judgment, so that he may be able to look upon things as they really are, and examine the subject from different sides and in various manners, before he takes up the pen. Many false statements have been made for the want of the proper qualification for accurate scientific research, and sometimes, I am sorry to say, have been made for the sole object of public notoriety, the common tendency of the day. In such cases, much honest labor is required to remedy the confusion, and to erase the false impressions made upon the medical mind by inconsiderate and erroneous statements.

It must be obvious to every rationally thinking mind, that for the mitigation or prevention of yellow fever, an intimate acquaintance with the pathology of this disease is absolutely necessary, and must form the basis of all inquiries or investigations, made in relation to its cause. It is for the general want of this thorough knowledge that so many speculative theories have arisen, concerning the nature of the cause of this disease. To obtain a more definite idea of the nature of the cause, however, is not the only object of acquiring a correct knowledge of the pathology of the disease, for there is another of more vital moment depending upon it, viz., its *rational treatment.*

Though in the more recent epidemics of yellow fever the treatment pursued by the better educated physicians has been more rational and less empirical than in former times, it cannot be denied that during an epidemic, when the demand for doctors is unusually great, a considerable portion of the medical forces called into requisition for treating and curing the many hundreds of cases, are but very imperfectly prepared for the proper discharge of such a duty ; this portion of the medical profession, however, might be better qualified if they were a little more acquainted with the true pathology of the disease, a knowledge upon which they could more safely rely in the selection of their remedies than upon too great confidence in their materia medica. And it is the earnest desire of counteracting the pernicious influence of quackery, prevailing to a considerable extent during each epidemic of yellow fever, and which cannot be but humiliating to the better part of the medical profession, that particularly prompts me to write this treatise. For this reason, no space will be wasted in a description of the history of the disease, or of the various epidemics which have prevailed in the different localities of the globe, for these subjects have been commented on so often as now to be familiar to every physician and layman. On the contrary, I shall confine myself to practical points, which directly bear upon the final mitigation or amelioration of the disease, and take the reader at once to the bedside, where he may study the clinical phenomena. From the sick-room, I shall take him to the dead-house to witness the macroscopical examination of the organs by the autopsy, and thence to the pathological laboratory, to obtain a knowledge of the microscopical pathological changes which, during the course of the disease, have taken place in the various tissues and organs. After being thoroughly acquainted with these facts, we are ready to enter into a comparison of the clinical phenomena with the pathological changes of the organs found after death, and synthetically to build up a general pathology, to be followed by a study of the probable nature of the cause, and the rational treatment of the disease. Lastly, after having thus studied and determined the character of yellow fever, and furthermore considered all points relating to it, we may venture to make a few remarks on its prevention.

### CLINICAL PHENOMENA, OBSERVED DURING THE COURSE OF THE DISEASE.

Most authors state that an attack of yellow fever rarely announces itself by any prodromal symptoms, but, in most cases, rather takes the patient by surprise. There are, nevertheless, many cases in which the fever is preceded by certain initiatory symptoms, similar to those observed in other infectious diseases, and consisting in a general feeling of uneasiness or discomfort, a want of the usual appetite, a certain degree of lassitude, more or less headache, etc., symptoms depending upon a general depression of the nervous system, and due to the action of the specific poison of the disease. In this condition the patient may remain for many days, especially when the disease prevails in the form of an epidemic, and when his mind is troubled with the cares and duties of family or friends, or by the general suffering and misery of which he is a daily witness,—or, he may succumb after the lapse of only a few hours. Nor is there any particular time for the insidious attack to take place. The patient may be surprised in the morning, or during the day, while engaged in business, or in the evening, and even during sleep. Although in most instances the disease appears suddenly, I am inclined to think that this phenomenon is more apparent than real, and, with the exception of very slight ephemeral cases, it is more probable that the fever is generally preceded by some prodromal phenomena, which, slight as they may be, announce its approach, especially in cases of a more severe and typical character. As the physician rarely observes the disease at the moment when the patient is first conscious of it, these forebodings, the prodromes, are usually overlooked—the patient himself not having been aware of them, as his mind was perhaps too much engaged with other matters. Thus, children are frequently attacked while playing with their toys, unconscious of the enemy who steals upon them and threatens their life; and, in many instances, the parents are not warned of the danger until they perceive the flushed face and heated skin of their child. It is generally accepted that in yellow fever, as in other infectious diseases, there is a period of incubation, which, commencing with the time of the absorption of the poison by the organism, may vary in length from a few hours to a number of days, or even weeks, during which time the poison, by increasing in quantity, is, so to say, getting ready for the attack. We may justly presume that such a period really exists, as there is no positive evidence that the fever ever suddenly appeared immediately after the exposure of the patient to the influence of the noxious poison; and, furthermore, that it appears quite improbable that the same poison should, during this whole period, remain dormant, without giving rise to any external phenomena indicating its presence in the system.

However slight or severe the prodromal phenomena may be, the real attack of the disease commences with the first symptoms of the febrile process. In most cases, perhaps, the disease is ushered in by an alternate sensation of chilliness and heat, associated with dryness of the skin; though many cases commence with a decided rigor. At the same time the patient suffers with pains in the head, back and limbs. The headache, which, to a slight extent, may already have existed as one of the prodromes, is chiefly confined to the forehead and temples—from which localities it may extend in an inferior degree over the rest of the head—and seems to be peculiar in character. The pain I experienced, even during a mere ephemeral attack of yellow fever, which I had, appeared to me more deeply seated than the neuralgic or rheumatic cephalalgia, from which I had occasionally suffered for a number of years; the sensation was more of a stinging or formicating nature. In most cases, the pyrogenetic or cold stage is of short duration, for soon the sensation of cold commences to alternate with one of heat, the latter predominating gradually over the former,—while a simultaneous rise of the temperature finally leads to the establishment of the hot stage.

Now, conscious of his state and confined to his bed, the patient presents an appearance of alarm and anxiety; he is restless, his face is flushed and his eyes appear muddy, owing probably to a slight œdematous swelling of the conjunctiva, produced by hyperæmia of that membrane. The tongue, at this stage, is, in many cases, found covered by a white, cream-like film, in others by a thin yellowish fur, while the point and edges remain free and are of a bright scarlet tint. As the disease progresses, the fever becomes more intense; the skin is very hot and dry; its temperature, though varying in different cases, always rising to 102–104° F. in the axilla. The pulse is full and hard, averaging from 100 to 130 beats per minute, the respiration hurried, frequently irregular. The pains in the head, back, muscles and joints of the limbs become more severe. The spinal pain, which is confined to the lumbar region, whence it may extend to the iliac portions, or to the whole of the pelvis, becomes almost intolerable, interfering with the rest of the patient, who, in seeking comfort, constantly changes his position in bed from one side to the other. Sometimes these symptoms are accompanied by gastric troubles, as nausea, and even vomiting of mucous or bilious matters, though, in general, the latter do not appear before the second or third day.

Accompanied by the above symptoms, the fever generally runs its course without abatement during the first day and night; but, severe as these symptoms may appear, there are, nevertheless, a considerable number of cases in which a favorable turn of the disease may already be observed on the day succeeding. In these cases the patient, usually after some free alvine and urinary evacuations, caused either by a simple effort of nature or by the effect of medicine previously administered, commences to perspire in the course of the night, and feels considerably relieved from his sufferings in the morning. At the same time, the pulse and temperature have considerably descended, so that before another night sets in the fever may have entirely subsided, leaving the patient in a state of nervous debility from which he may soon recover. These cases represent the *ephemeral* form of yellow fever, accompanied from its first appearance by violent symptoms, which, however, speedily subside without leaving any serious traces behind. My own case was one of this kind.

But, in the majority of cases, the fever does not abate so soon, but continues to the third or fourth, sometimes even to the fifth day. The temperature, then, may still rise higher, to from 105 –107° F., in exceptional cases even higher, while the fall of the pulse, ordinarily commencing on the second day, generally reaches the normal standard on the fifth or sixth day, and frequently even descends still lower. Gastric trouble, also, consisting in nausea, and a constant inclination to vomiting, if not present already, now commences. Generally, there is a sensation of a dull aching pain, or pressure in the epigastric region, arising from the irritation of the mucous membrane of the stomach, and caused by the now beginning hyperæmia of this organ; the nausea and inclination to vomit are also due to this condition. This painful sensation, however, is not in all cases increased when pressure is made by the hand upon the epigastrium. Still, the nausea, and the constant, often unsuccessful attempts to vomit, are very annoying, causing much suffering to the patient. If he succeed in his efforts to throw up anything from the stomach, which at this time is generally empty, the vomited matters are found to consist of a thin mucoid, sometimes ropy fluid, of a white and frothy appearance, frequently mixed with bile or small portions of undigested food; in some cases it is even mixed with pure bile, presenting, then, a green color. A microscopic examination reveals that this fluid is principally loaded with large flat epithelial cells, belonging to the scaly or squamous variety of epithelium, not derived from the epithelium of the stomach, but from that of the mouth, pharynx and œsophagus. Many of them are floating singly, but the greater part, adhering to each other in their former positions, still form small shreds of epithelium of different sizes. Others, again, are in a state of disintegration; their outlines have become faint, showing that their protoplasm is about to dissolve, resulting in the setting free of their contained nuclei and granules. It is from this source that the free nuclei and numerous granules floating in the liquid are derived. In some specimens of this frothy mucoid matter I have met with zoglœa of micrococci, or with other bacteria; in other instances, however, these organisms were absent. In correspondence with the presence of the large epithelial cells, derived from a scaly epithelium, it will be found that by examining the fauces and pharynx, these parts, in some instances also the tonsils, are considerably congested, and I entertain no doubt that frequently the congestion extends farther down into the œsophagus. During the epidemic of 1867, I met with one particular case in which any liquid which the patient attempted to swallow never entered the stomach, but was invariably rejected before it passed mid-way of the œsophageal tube, the phenomenon depending very probably on a temporary stricture, produced by the swelling of the mucous membrane of the œsophagus; the difficulty was relieved by the application of a warm poultice. From the above examinations it may be presumed that the epithelial elements are surely derived from the congested parts, while the mucoid liquid may come from the stomach.

There are a number of cases, in which these gastric phenomena accompany the fever from its first appearance, but, generally, they manifest themselves on the second or third day. They should always be closely watched by the physician, and never be overlooked in the prognosis of the case; for the relation they bear to the final issue, in indicating the condition of the liver, as I shall show hereafter, is as important as that of the temperature, indicating the intensity of the fever.

The congestion of the conjunctiva, to which we ascribed the muddy appearance of the eyes on the first day, has now become more or less developed, giving to these organs a moist appearance. The fur of the tongue has increased in thickness, and has assumed a yellow or brownish tint. The patient is very thirsty, and constantly demands cold water to drink. This excessive thirst is usually referred to the disturbed circulation of the mucous membrane of the stomach, though I think that the fauces, pharynx and œsophagus may contribute their full share. Frequently, the water, after having remained a short time in the stomach, is thrown up again. The urine, which at first was natural in appearance, assumes now a more reddish tint, and, in some cases, it may be found to contain some albumen, even at this early period. The bowels are usually costive. Quite frequently the patient complains of pain in the lower part of the abdomen, directly over the region of the urinary bladder, indicating that this organ also participates in the congestion generally observed in the mucous membranes, or in other secreting organs, a condition which I have found verified in a few instances by post-mortem examination. Fortunately, the congestion in the bladder is usually slight, the pain being soon relieved by the application of warm poultices, and often followed by a passage of urine. In many cases, the perspiratory glands of the skin have now resumed their function—in some instances to such a degree as to become excessive. Sometimes the perspiration is so profuse as not only to completely wet the patient's clothes, but also the bedding upon which he rests. It is a singular fact, which attracted my notice during the epidemic of 1867, that however profuse the perspiration might be, there was no abatement in the fever observed, the temperature of the skin remained the same, or even rose, while the pulse continued to beat with the same force as before. At this time, also—sometimes earlier, but generally when perspiration commences—a peculiar odor is perceived to emanate from the patient, and, moreover, a peculiar burning, stinging sensation is imparted to the points of the fingers when the moist and heated skin is touched, as is done in feeling the pulse. I have frequently felt this stinging sensation, after I had washed my hands and left the patient. The peculiar odor emanating from the skin, and which, according to some authors, is also perceived in the breath, has been noticed by a number of physicians, and by some even been regarded as pathognomonic of yellow fever, while others have not been able to detect it. The failure of the latter, however, may be due to their sense of smell not being sufficiently acute, a circumstance rendered more than probable in consideration of the greater or lesser difference surely existing with regard to the other special senses among different individuals. I can entertain no doubt of the existence of this odor, for I have perceived it distinctly, and without a previous knowledge of it, during the epidemic of 1867 and afterward; and it is also observed by the friends and nurses of the patient. But that a particular diagnostic value should be attached to it, I

would not venture to assert in face of the fact that similar odors are perceived in other acute infectious diseases, as small-pox, scarlet fever, measles, etc. In small-pox, the odor is very distinct, and may, as I know from experience, even be perceived before the eruption makes its appearance ; to myself, however, a difference appears to exist between the odors of the two diseases.

The most important clinical phenomena, observed in the whole course of this disease, are certainly those arising from the noxious influence of the specific poison upon the nervous system. In fact, the nervous symptoms are, perhaps, the first by which the disease announces its approach, and are therefore prominent among the prodromes, manifesting themselves in the form of the slight headache and the general depression already referred to. When the real disease breaks out, these symptoms become more developed, and take a part in the chill or rigor; finally, however, after the commencement of the fever, they show themselves again in the restless condition of the patient. The severe pains in the head, spine, joints and muscles, and even the occasional spasms in the latter, especially indicate a strong afflux of blood to the cerebro-spinal axis, terminating, in most cases, in the establishment of a true hyperæmia, not only of the brain, but also of a portion of the spinal marrow ; and, furthermore, as I shall show hereafter, of the thoracic and abdominal sympathetic ganglia. The steady progress of the hyperæmia is easily recognized by the pain in the head and spine increasing in severity, and giving rise to sighs and groans on the part of the patient. In correspondence with this condition, therefore, a slight delirium may in a number of cases be observed as early as on the first day ; if not, it surely makes its appearance on the second, though it may be of a very mild character. In mild cases, it may only consist in irrational language or mutterings, uttered by the patient as he restlessly turns from side to side in a state of sleepy wakefulness ; a state from which he may, however, be easily aroused when spoken to, and even return a rational answer. But, in many cases, the delirium becomes more severe, and frequently attains such a degree as to render the patient violent, necessitating the use of force to restrain him to his bed, and to protect him from injuries which he might inflict upon himself. In fatal cases, delirium is rarely absent during the course of the disease.* I am inclined to think that, with a very few exceptions, the fatality of the case always depends upon the hyperæmia of the brain.

Under these circumstances the second day and night pass away, and the third day arrives, when, perhaps in most cases, the febrile storm commences to subside. At the same time, the accompanying symptoms have also considerably abated, and the patient, greatly relieved from his pain and anxiety, feels comparatively comfortable. In many cases, however, the fever does not subside until the fourth or fifth day. Then, the above described symptoms continue with greater or lesser intensity, with the addition of others, such as temporary retention of urine, costiveness, etc. The urine, also, almost invariably now contains albumen. Frequently, the febrile symptoms commence to abate soon after a free evacuation of the bowels or bladder. With the abatement of the febrile process, the temperature also begins to fall, and the pulse, which, in most cases, commenced to fall as early as on the second day, continues its descent in the same proportion. Some slight cephalalgia, or some tenderness over the epigastric region may still exist. In favorable cases, convalescence now commences and may continue without any serious interruption, until the patient has been restored to his former health.

But, unfortunately, the disease does not run in all cases the favorable course above described. On the contrary, only too often, after the subsidence of the febrile storm, graver symptoms, more or less typhoid in character, appear. In such cases, the patient, as in those above described, though prostrated from the fever, feels, also, comparatively easy and relieved from his sufferings at the end of the febrile stage ; very often the relief is so great as to induce him to think the disease has left him entirely, and that in a few days he will be able to resume his daily occupation. This condition, representing the so-called second stage, is very delusive, and may last from two to three, or twenty-four hours, sometimes even longer, when the symptoms return in another form, and the patient approaches the third stage of the disease. The fever then lights up ; the temperature which had commenced to fall, may rise again ; the pulse also becomes accelerated, but instead of being strong and full, as in the commencement of the disease, it is now small and thread-like. The irritability of the stomach has increased, and is again accompanied by the vomiting of a mucoid fluid mixed with particles of food previously taken, sometimes also with flakes of black hæmorrhagic matter, or even with pure blood. The act of vomiting, however, is not performed with the same violence as before; the matters are easily thrown up. The stomach rejects anything in the shape of food or drink. At this time, also—though frequently earlier—the conjunctiva commences to present a yellowish tint, which, as the disease advances, increases in intensity. The same tint is observed in the scanty urine, which now almost invariably contains albumen. A microscopical examination of this fluid frequently shows small granular masses, and according to some authors, albuminous casts besides. The skin, especially that of the face, neck and shoulders, also assumes the yellowish tint, which, as the disease progresses, gradually extends downward over the whole body. In many instances, hæmorrhages occur from the nose and gums, and even from the ears and the conjunctiva. The tongue is generally dry, and the yellowish fur covering it during the first stage of the disease, has now assumed a dirty brownish tint; the edges, if clean, are, together with the gums, of a purplish color. The nervous symptoms are rendered more prominent; the patient becomes again more restless, and the delirium returns; but as the patient's strength is now wasted, the latter has lost part of its violence, though, sometimes, it retains its character furious up to death. Frequently, muscular spasms of the limbs and subsultus tendinum occur. As the vomiting continues, the flakes of black hæmorrhagic matter gradually increase in amount, until at last a large quantity of them, mixed with a thin mucoid fluid, and known as "black vomit," is ejected at once. Often, with the ejection of this fluid, the violence of the symptoms is reduced, and the patient, though comparatively prostrated, feels relieved from the sensation of pressure, or tightness over the epigastric region.

Before continuing our sketch of the clinical symptoms, it may be proper to first describe the microscopical character of the "black vomits." When a fresh specimen of black vomit, ejected from the stomach of a severe case of yellow fever, is, for a short time left standing in a bottle, it separates into two parts, the one of which represents a thin mucoid fluid, while the other, consisting of its solid parts and resembling coffee-grounds, settles to the bottom of the vessel. The mucoid fluid represents most probably the water with the liquid food, previously introduced into the stomach of the patient, mixed with the mucous secretion of that organ. The solid matters of the vomit, bearing a more important relation to the pathology of the disease than the fluid, consist, besides the disintegrated matters of food, such as fat, muscular fibers, etc., mainly of the constituents of blood and epithelium. A part of these constituents of the blood is represented by a considerable number of blood corpuscles, which, however, are now deprived of their coloring material, the hæmoglobin, appearing, therefore, perfectly colorless. They are characterized by a delicate though well defined double contour, and, in some cases, have preserved their normal diameter, while in others they have more or less decreased in size. In all the numerous specimens of black vomit which I have examined, the blood corpuscles were always found colorless, without regard to their diameter. This phenomenon is easily explained in considering the facility with which these bodies part with their coloring material, when surrounded by a fluid of a lower specific gravity than that of the liquor sanguinis. The water with which the patient allays his thirst, and of which a larger or smaller quantity is almost always present in the stomach, is alone sufficient to deprive the blood corpuscles of their hæmoglobin; though it is very probable, as will be seen hereafter, that these corpuscles part with a portion of their coloring matter while still contained, in a state of stasis, in the interior of the minute vessels, and before a rupture of the walls of the latter may have occurred. The other part of the constituents of the blood, found in the vomit, consists of the free hæmoglobin, or hæmatin, presenting itself in the form of yellow amorphous patches. Generally, these patches are found in company with smaller or larger groups of blood corpuscles, or even enclosing them, from which circumstances it may be presumed, that the escape of the coloring material from the corpuscles occurred already in the interior of the vessel, and during a state of stasis. In some specimens, the yellow patches are observed to contain minute floccules, or masses of organic granules, probably derived from the mucous membrane of the stomach. Besides these elements, a number of colorless blood corpuscles, and free epithelial cells, or their remains, are also met with.

In order to prove that these yellow patches in reality represent the coloring matter of the blood corpuscles, instead of bile, as has been stated by some observers, I filtered a quantity of fresh black vomit, and allowed the solid parts remaining behind upon the filtering paper to dry, for the purpose of applying the well known test of *Teichmann.* Accordingly, I placed a small portion of the dried material—about the size of a head of a pin—upon a glass slip, and, after rubbing it up with a trace of table salt into a fine powder, put a covering glass upon it. A drop of glacial acetic acid was then allowed to run under the latter, and the whole preparation gently warmed over the flame of a spirit lamp, until small bubbles of gas were observed to be formed. A microscopical examination, made after the preparation had cooled, showed a number of minute crystals of the muriate of hæmatin, the so-called *hæmin* crystals of Teichmann, universally regarded as the surest proof of the presence of hæmatin. In some specimens of black vomit, however, I have found hæmatin crystals already formed.

Almost in all specimens of black vomit, certain fungous growths are met with. The one most frequently found is the yeast plant, or *Cryptococcus cerevisiæ.* If the specimen is left standing, other species of fungi will be developed. Very often, also, colonies, or so-called *zoglœa of micrococcus* are observed in fresh specimens; free oscillating sphero-bacteria, however, are rarely seen, unless the fluid has been standing for some hours, or longer. Some importance has been attached to the presence of these organisms in black vomit, and attempts have been made to establish a relationship between them and the cause of yellow fever. In the beginning of the epidemic of 1867, the germ theory, which, not long before, had commenced to attract the attention of medical men, had also taken root in my mind, inducing me not only to search for the germs in the very numerous specimens of black vomit which I then examined, but, moreover, upon the various mucous membranes of the body in the numerous autopsies which I made. Failing, however, in my attempts to discover anything in support of this theory, I did not hesitate to discard it; for, as regards the fungi met with in the black vomit, it is obvious that the germs, from which they are derived, reach the stomach with the food and drink, and with the saliva of the patient, where they undoubtedly find the necessary conditions for their development.

In continuation of our sketch of the symptoms observed in the third stage of the disease, we may state that severe and dangerous as they appear to be, and really are, they do not necessarily determine the death of the patient. On the contrary, even at this period, the disease may still take a favorable turn. In such a case, the fever, which may have lasted about twelve hours, —rarely more—gradually subsides, and with it all other symptoms assume a milder character. The skin becomes moist, the pulse falls again, though remaining very feeble and compressible. The gastric symptoms have greatly diminished, the vomiting has ceased, and only some occasional eructations may be observed. The temperature, though still above 100° F., has fallen considerably, the defervescence continuing until convalescence is established. The delirium also passes, leaving behind a moderate cephalalgia. The most prominent symptom now left is the great nervous prostration. The nervous energy of the patient has been wasted, and all that remains scarcely suffices for the ordinary supply of the various organs of the body. Nevertheless, with all this damage, the patient may rally, and with the proper nursing, and careful treatment, convalescence may be established, though, in most of these cases, it is tardy, and often accompanied by other complications, such as diarrhœa, abscesses, periostitis of the tibia, suppurative parotitis, etc. I have seen a number of cases recover after black vomit had occurred, particularly children.

If, on the other hand, no amelioration of the disease occurs in the beginning of the third stage, the symptoms above described assume a character still more grave. The temperature rises still higher, while the pulse often sinks, sometimes to rise again before death. Not only more black vomit is thrown up from the stomach from time to time, but, in some cases, also, the same black matters are passed from the bowels. The urine becomes very scanty, sometimes suppressed for many hours—from twelve to twenty-four, or even forty-eight hours; and if voided, it is in an abnormally small quantity, and muddy in appearance. In exceptional cases, the suppression of urine lasts until death. The hæmorrhages from the nose, gums, fauces and tongue become more frequent, and others, from the kidneys and bladder, may take place, the blood escaping from the urethra. The tongue is hard and dry, covered with a brown or blackish fur, sometimes scabby; the gums are swollen, and of a purplish color, frequently covered with sordes. In many cases hiccough appears; the patient now approaches collapse.

In a number of cases, especially those terminating fatally in the early part of the third stage, the delirium remains active until death approaches. Then, the patient is very restless, constantly attempting to rise, and if succeeding, soon falls back upon the pillow, when life may be suddenly extinguished; while, in other instances, the delirium continues in a low and muttering form until death.

In cases where the third stage extends over several days, the symptoms, already mentioned, continue alternately with more or less violence until collapse sets in. The patient then breathes with difficulty, sending forth deep sighs and moans; often, his face is of a dusky color, though the whole skin has assumed an orange-yellow; his countenance is sunken, his tongue tremulous. He lies there, with dry, scabby lips, and frequently with a bloody mucoid liquid escaping from the corners of his mouth; indifferent to, or, more truly, unconscious of his condition, muttering incoherent sentences, until death relieves him from his sufferings.

The above sketch of the clinical symptoms, observed in the course of yellow fever, as it occurs in the average number of cases, I do not consider entirely complete; some additions will, therefore, be demanded to fill up the vacant spaces, though not to such an extent as to embrace all the various exceptional forms in which the disease may appear, and which mainly depend upon the age, sex, temperament, peculiar predisposition and other previously existing conditions of the patient.

Thus, as regards the temperature, it remains to be mentioned that certain regular fluctuations are observed, consisting in slight evening exacerbations and morning depressions; but, these may correspond with the diurnal fluctuations of temperature observed in health, and they cannot be looked upon as regular remissions; especially as they are equally observed in other "continued" fevers. Although during the epidemic of 1867, and at other occasions afterward, I made use of the thermometer for the purpose of estimating the intensity of the fever, it was done without regularity and system, and no notes were taken from which accurate deductions might have been made. To the extent of my knowledge, it was by *Dr. J. C. Faget*, of this city, that the first systematic observations were made regarding the rise and fall of the temperature of the body, as it occurs during the course of yellow fever; and, judging from the results of his observations, together with those of other observers, the elevation and depression of heat, and the rise and fall of the pulse during the febrile process, appears to take place after a certain type, pathognomonic to this disease. In a treatise, entitled "*The Type and Specificity of Yellow Fever, etc.*," published in 1875, Dr. Faget presents the rise and fall of the temperature and pulse—marked by dots and lines, upon systematically arranged charts—of thirty cases of yellow fever, collected and prepared by himself, Drs. *Tuatre* and *Layton*; and, furthermore, the charts of seventy-three cases, prepared from the tables of *Dr. Dudley S. Saunders*, published in his article, entitled "Observations on the Yellow Fever of 1873. Epidemic at Memphis, Tenn."* The deductions Dr. Faget made from his observations were: "That the course of the temperature in yellow fever, during the epidemic of 1870 in New Orleans, was characterized by a *single paroxysm*, the appearances of which, from one to three days in duration, was followed, without a stationary stage, by a defervescence of from four to seven days." Regarding the pulse, he states: "The maximum average being 120 pulsations on the first day; on the second day the pulse beats ten or twelve times less; in another twenty-four hours there are a dozen pulsations fewer;

* New Orleans *Medical and Surgical Journal*, May, 1873.

the *fall continues* on the following days, but with a *progressively decreasing rapidity;* in general, on the fourth day, the pulse oscillates toward eighty, and, with this figure, it has still hardly reached half of its return course, which will be completed only toward the seventh or eighth day; the descent will then go on still further, and toward the tenth day an astonishing *minimum* will be occasionally obtained; in some patients in convalescence, the pulse does not exceed forty in a minute." The average figures of the Memphis charts correspond in the main with those of the charts of Dr. Faget, and his statements, regarding the temperature and pulse in yellow fever, appear to be further corroborated when compared with the tables published by *Dr. Jos. Jones*, and prepared from his own records of ninety-three cases, and from the dates, obtained at the Charity Hospital by some of the resident students, of ninety-five cases.* Slight deviations depending on various causes, of course, may be observed to occur in different cases; yet, on the whole, the rule, laid down by Dr. Faget, seems to be true, and, if furthermore confirmed by numerous additional observations, will greatly assist in the diagnosis of the disease, even as early as the second day, when the pulse commences to fall, while the temperature rises.

Much importance has always been attached to the interruption or failure of the urinary passages during the course of yellow fever; that is, at a time when, under ordinary circumstances, they should appear. And it is not only the physician who is anxious to see the kidneys and bladder continue to perform regularly their functions, but the nurses and friends of the patient, also, knowing from hearsay the danger associated with a suppression of urine, watch the performance of the urinary function with interest and anxiety, and seldom fail to report on this subject to the physician, if he should forget to make his accustomed inquiries. Suppression of urine, it is true, is one of the most dangerous symptoms met with in this disease, and, if not relieved, will be followed by the death of the patient. But, I am inclined to think, that in many cases in which the non-appearance of the urine is ascribed to a suppression of the func-

* *Jones*—"Yellow Fever Epidemic of 1878 in New Orleans." New Orleans *Medical and Surgical Journal*, June, 1879.

tion of the kidneys, this secretion is only retained, small as it may be in quantity, in the bladder. To this view I have been led by my macroscopical and microscopical examinations of the urinary bladder and kidneys. Whenever a suppression of urine really occurs, it is always during the latter part of the third stage of the disease, and in protracted cases, after sufficient time has elapsed for the destructive pathological changes in the parenchyma of the kidneys to take place. A temporary retention of urine often occurs during the first stage of the disease, when it may depend on an irritation of the mucous membrane near the outlet of the bladder, exciting the muscular fibers of the sphincter to contraction. The relief obtained by the application of warm poultices over the region of the bladder seems to corroborate this view. If the retention occurs after the febrile stage, when the nervous energy of the patient is greatly depressed, the phenomenon may be owing to a debility, or even temporary paresis of the muscular element of the bladder itself. That such a condition may exist becomes very probable in considering the spinal hyperæmia in the lumbar region, the existence of which is not only indicated by the excessive pain in this region during life, but, moreover, confirmed by the hyperæmia, observed after death in the pia mater of the spinal marrow of the same region, extending even to the marrow itself. The details of this subject, however, I shall discuss more fully in connection with the pathology of the disease.

In regard to the "black vomit," it should be mentioned, that not infrequently fatal cases of yellow fever are met with, in which this clinical phenomenon does not manifest itself, though all other characteristic symptoms of the third stage, indicating that a hæmorrhage from the mucous membrane of the stomach must probably have taken place, may be present. An autopsy, made in such cases, will invariably reveal the presence of a larger or smaller quantity of the same black matters in the stomach; sometimes also in the intestines. The retention of these matters in the stomach during life is obviously owing to an excessive debility, not only of the muscular coat of that organ, but of all other muscles concerned in the act of vomiting, corresponding to the general nervous exhaustion of the patient.

Although our description of the symptoms accompanying yellow fever is applicable to the great majority of cases, which during an epidemic come under the observation of the physician, there are nevertheless a number of cases met with, in which the symptoms do not present themselves in the general order as above described. Such deviations from the general type, however, will always be found to depend on peculiar conditions, predispositions, or even idiosyncracies of the patient, modifying the ordinary course of the disease. To regard these various modifications as separate forms of the disease, as has been done by a number of authors, would, indeed, render the whole subject almost too complicated for the practicing physician; and it must, therefore, be left to his fundamental knowledge and sound judgment to distinguish between these forms, and, accordingly, shape his treatment. A mere distinction between a sthenic and an asthenic, or adynamic form, would suffice for all practical purposes.

A peculiar form of yellow fever, termed by La Roche the "walking grade," has been mentioned by authors, and is frequently spoken of by physicians, though I have never met with it myself. In this form, using La Roche's description, "the patient, though sometimes in bed, is found more frequently sauntering about his room; and, indeed, he at times walks about the street for recreation or business; and though, in some instances, he states that he is weak, in others he exhibits at intervals, or throughout, marks of considerable muscular strength. He complains of nothing, denies his being ill, amuses himself in reading or otherwise, and, to a casual observer, appears to be slightly, if at all, indisposed. To the physician, however, matters appear in a different light; for he may gradually observe that the patient exhibits an unusual expression of countenance—dull and listless. The eye is watery; the complexion is almost mahogany color, while the pulse is found exceedingly weak, and even totally absent. Black vomit overtakes him, even while occupied in the way mentioned, or very soon after, and death speedily ensues."

In considering the symptoms, mentioned in the foregoing description, they are easily recognized as belonging to the second or third stage of the disease; and by a closer inquiry into the circumstances of such cases, it would most probably be found, that the patient, though really feeling ill, by some peculiar notion or caprice of his own denies this fact, while by the strength of his will he manages to walk about, and attend to his daily occupation. We may further presume, that a febrile stage has previously existed, though in a very mild form, enabling the patient to deceive himself and his friends; and, moreover, that the poison of the disease, while affecting the brain very slightly, impressed its noxious influence more upon the sympathetic ganglia, affecting the heart, liver and stomach in such a degree as speedily to interfere with the functions of life.

As a counterpart of these "walking cases," those may be mentioned in which the patient, by an extreme fear of the disease, exhausts his nervous energy, and reduces his system to a condition most favorable to the reception of the noxious poison. A remarkable case of this kind I met with in my own practice during the epidemic of 1870. It was a young man, a German, of about twenty-three years of age, who consulted me at my office, believing himself attacked by the yellow fever. By examination, I found him apparently in good health, with no symptom of any fever about him capable of detection by the touch, as the thermometer was not used. Notwithstanding, I failed in my endeavors to convince him of this fact; and my advice, to put his mind at rest by dismissing his gloomy thoughts, made no impression upon him. He left my office, and before night sent for me to visit him at his room. I found him in bed, and in the same apparently healthy condition. No symptom of disease could be discovered, but he was still deaf to the encouragement he received from his friends and myself, calculated to dispel his fears and arouse his spirit. The next day, during which he took but little nourishment, passed in the same manner; but, on the third day, fever appeared, which, however, never became excessive. On the contrary, refusing all nourishment, and evincing a considerable apathy for everything around him, he gradually passed into a lethargic condition from which no moral or physical stimulants could arouse him. On the fourth day, tenderness by pressure upon the stomach was detected, followed by black vomit and symptoms of collapse in the evening; he died during the night in a state of coma.

Although in this case no external symptoms of the disease could be detected during the first two days, we are nevertheless justified in presuming that the poison primarily affected the mind of the patient, impressing him with the idea that something was wrong with him, and then slowly exerted its noxious influence upon the abdominal organs, and probably also upon the heart.

Another form of yellow fever, mentioned by La Roche, is the "apoplectic grade." In cases of this kind, "the patient," as this author states, "is struck down suddenly, as if by lightning, with stupor or coma, and death, preceded by convulsions, soon follows. In other instances, the progress is less sudden. Without even the slightest premonitory symptoms, the patient is in an instant seized with vertigo and confusion of mind. He complains of dull pain and fullness of the head, together with spasmodic pain and considerable debility in the legs; coldness, debility, and a feeling of uneasiness in the spinal region. The pulse varies in different cases in point of fullness and frequency, but is always weak, and finally becomes faltering. The skin is cold, sometimes dry and flabby, but generally unctuous or bedewed with cold perspiration; the stomach is sometimes irritable. In the meantime, the patient lies as if stunned, with dilated pupils, and an expression of gloom on his countenance. From this unpromising state an effort of reaction occasionally takes place, but this scarcely ever leads to a successful result. More generally, the patient becomes perfectly comatose; the eyes assume a glassy appearance, the pulse fades away, involuntary discharges and profuse hæmorrhage supervene, and death soon ensues."

In considering the symptoms of these so-called "apoplectic" cases, I cannot but think, that, perhaps in all of them, a diseased condition of the anatomical elements of the brain, especially of its minute blood-vessels, has existed previously to the attack of yellow fever. This view is corroborated by my discovery of fatty degeneration of the nuclei of these vessels in a number of patients who died from yellow fever. In face of the fact, however, that a rupture in the walls of the minute veins of the mucous membrane of the stomach really does take place in the course of this disease, I would not venture to deny such an occurrence in the brain, giving rise to the capillary form of apoplexy. But as, to the extent of my knowledge, there is no record of apoplectic foyers discovered in the brain after death from yellow fever, I presume that the symptoms attending these "apoplectic" cases may be rather attributed to a hyperæmia of that organ, of a very high degree, together with a direct action of the noxious poison upon the substance of the cortex cerebri itself.

In viewing in this light the various forms of yellow fever, described by a number of authors, it becomes obvious that they are equally produced by one and the same poison, affecting the organism under different conditions, but always accompanied by one or more of those symptoms characteristic of the disease. None of these forms bears a peculiar, distinctly defined character; for, if this were the case, it might also be presumed that each separate form of the disease depended on a separate form, or state of the poison. Whatever, therefore, the conditions of the organism may be when attacked by yellow fever, the latter will always show its own and distinct character, expressed by its pathognomonic symptoms during life, and also by the pathological condition of the various organs, observed after death.

When yellow fever supervenes upon another pre-existing disease, it may modify the accompanying symptoms of the latter, though it will always impress upon it its own peculiar marks. In another section of this treatise, I shall speak of a case bearing upon this subject.

[TO BE CONTINUED.]

